# Enlargement of a Modular System—Synthesis and Characterization of an *s*-Triazine-Based Carboxylic Acid Ester Bearing a Galactopyranosyl Moiety and an Enormous Boron Load

**DOI:** 10.3390/molecules24183288

**Published:** 2019-09-10

**Authors:** Martin Kellert, Peter Lönnecke, Bernd Riedl, Johannes Koebberling, Evamarie Hey-Hawkins

**Affiliations:** 1 Institute of Inorganic Chemistry, Faculty of Chemistry and Mineralogy, Leipzig University, Johannisallee 29, 04103 Leipzig, Germany (M.K.) (P.L.); 2 Bayer AG, Aprather Weg 18A, 42113 Wuppertal, Germany (B.R.) (J.K.)

**Keywords:** boron neutron capture therapy, carborane, *s*-triazine, cancer therapy, modular system

## Abstract

The amount of boron accumulated in tumor tissue plays an important role regarding the success of the boron neutron capture therapy (BNCT). In this article, we report a modular system, combining readily available starting materials, like glycine, 1,3,5-triazine and the well-known 9-mercapto-1,7-dicarba-*closo*-dodecaborane(12), as well as α-d-galactopyranose for increased hydrophilicity, with a novel boron-rich tris-*meta*-carboranyl thiol.

## 1. Introduction

Since the early 1950s, boron neutron capture therapy (BNCT) is regarded as a very promising method for cancer treatment [1,2]. The binary therapy uses non-toxic components, boron-10-containing reagents and thermal or epithermal neutrons, to produce cytotoxic species, which are able to destroy malignant tissue. Boron-10-containing drugs linked to a tumor-selective functional group to address the cancer site are highly advantageous [3,4,5,6,7,8,9,10]. Ideally, the non-toxic bioconjugate shows selective accumulation and retention in the malignant tissues in the required amount of 10–30 µg/g tumor and can, subsequently, be irradiated with thermal or epithermal neutrons [3,11,12]. It depends on the drug carrier if the BNCT agent is just accumulated in the tumor tissue or internalized into the cancer cells; internalization increases the efficacy of this cancer treatment [10,13,14,15,16,17,18,19,20,21,22,23,24,25,26,27]. Following the neutron capture event, the generated particles are lithium and helium nuclei (α particles) and possess a high linear energy transfer (LET) [3,28,29]. These high-energy particles with a mean free path of about 5 to 10 µm reveal their destructive action only in a limited area [3,8,9]. Although the combination of suitable boron-rich molecules with tumor-selective moieties allows a very selective tumor treatment, which only affects malignant and spares normal tissue [13,15,17,23,24,25,30], there are still some relevant challenges, including, but not limited to, the selectivity of the chosen biomolecules for a specific tumor type, the required boron-10 concentration in cancer cells, the water solubility of the final bioconjugate, and the neutron beam quality [31], which are the focus of recent studies [16,25,26,27,32,33,34,35,36,37].

**Figure 1:**
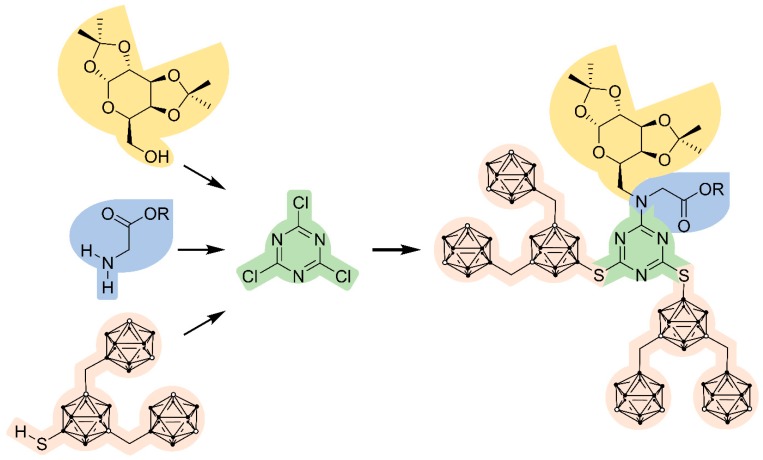
Illustration of the modular system incorporating an α-d-galactopyranosyl moiety (yellow), protected glycine (blue), a tris-cluster derivative of 9-mercapto-1,7-dicarba-*closo*-dodecaborane(12) (pale red) and cyanuric chloride (green) as core; R = *tert*-butyl.

Due to the necessity of high boron concentrations in malignant tissue, research for BNCT drugs with a high boron content is highly demanded [13,14,15,17,23,38,39,40,41,42]. There are already many strategies employed to develop specific shuttle systems; however, some are associated with undesired cytotoxic side effects (polycationic compounds) [42], low yields in synthesis (encapsulation of boron compounds in liposomes) [13], low selectivity (cell-penetrating peptides [17], dendrimeric BNCT drugs [14]) or deboronation reactions (application of *ortho*-carboranes) [43,44]. Recently, we reported the synthesis of a modular system to prepare potential precursor molecules for novel BNCT agents [45,46,47]. Here, we describe a modified modular system for the preparation of a carboxylic acid derivative with a very high boron load (Figure 1) suitable for coupling with a variety of biomolecules.

## 2. Results and Discussion

The application of carboranes with nucleophilic carbon atoms, like carboranyl lithium compounds or Grignard reagents obtained from bromomethyl-*ortho*-carborane, is widespread [48,49,50,51,52,53]. For C–C bond formation, the Kumada coupling reaction between Grignard reagents and organohalides with palladium catalysts is very useful [54,55,56], and can also be extended to carborane derivatives. Thus, Kumada-like reactions are known for 9-iodo-1,7-dicarba-*closo*-dodecaborane (**1**) and even for tetraiodinated derivatives [57,58,59], and are also employed here for the preparation of boron-rich derivatives.

**Scheme 1:**
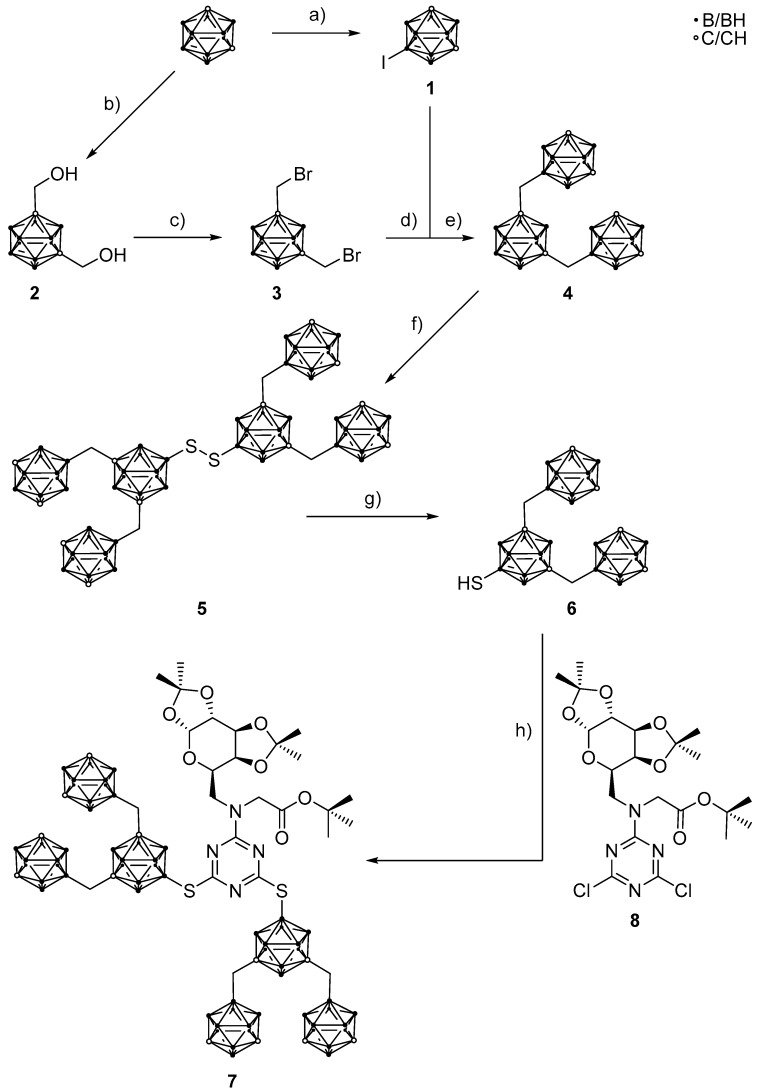
Preparation of *tert*-butyl-*N*-{4,6-bis[(1,7-dicarba-*closo*-dodecaboran-9-ylmethyl)1,7-dicarba-*closo*-dodecaboran-9-ylthio]-1,3,5-triazin-2-yl}-*N*-(1′,2′:3′,4′-di-*O*-isopropylidene-6′-deoxy-α-d-galacto-pyranos-6′-yl)glycinate (**7**). a) I_2_, AlCl_3_, CH_2_Cl_2_, rt, 2 d, 92% [62,63]; b) paraformaldehyde, *n*-BuLi, tetrahydrofuran (THF), rt, overnight, 89%; c) Br_2_, PPh_3_, benzene, reflux, 46 h, 95% [64,65,66]; d) from **3**—Mg, THF, reflux, 2 h; e) from **1**—11 mol% CuI, 4 mol% [PdCl_2_(PPh_3_)_2_], THF, reflux, 2 d, 30% [61,62,63,64,67,68]; f) S_2_Cl_2_, AlCl_3_, CH_2_Cl_2_, reflux, 4 h, 67% [69]; g) Zn, HCl_conc._, glacial acetic acid (HOAc), ethyl acetate, reflux, 4 h, 81% [69]; h) +**8**, K_2_CO_3_, MeCN, reflux, 2 d, 79%.

The iodination reaction of *ortho*- and *meta*-carboranes at the 9-position is well-known [60,61,62,63,64], so the synthesis of the corresponding derivative **1** was straightforward (Scheme 1, step a). For the synthesis of the bromomethyl derivative **3**, based on the metabolically stable *meta*-carborane, only a few examples are known [65].

According to the literature, the synthesis of 1,7-bis(hydroxymethyl)-1,7-dicarba-*closo*-dodecaborane(12) (**2**) [66] was straightforward (Scheme 1, step b), and bromination of both hydroxyl groups was carried out following the procedure for the mono-substituted derivative 1-hydroxymethyl-1,7-dicarba-*closo*-dodecaborane(12) [65], giving 1,7-bis(bromomethyl)-1,7-dicarba-*closo*-dodecaborane(12) (**3**) in excellent yield (Scheme 1, step c) (analytical details are given in the electronic supporting information).

After the successful synthesis of **3** and **1**, the following Kumada-like coupling reaction (Scheme 1, steps d or e) was carried out. The coupling of two carborane clusters via a methylene group with palladium catalysts has already been reported [68,69]; however, the tris-*meta*-carborane moiety **4** obtained here with a large boron content of 30 boron atoms per single molecule is new (Scheme 1).

After work-up, **4** was isolated in 30% yield [56,62]. According to the proposed mechanism for the copper(I)-assisted Kumada coupling, some of the Grignard reagent is used to activate the palladium catalyst, and therefore, undergoes a homocoupling reaction, which can be the reason for low yields [62]. However, here, this reaction has only a marginal impact on the yield, and the major side products are the result of the incomplete conversion of **3** or the respective Grignard derivative. The observed side products after work-up, namely 1-(hydroxymethyl)-7-(1,7-dicarba-*closo*-dodecaboran-9-ylmethyl)-1,7-dicarba-*closo*-dodecaborane (**SP1**) and 1-methyl-7-(1,7-dicarba-*closo*-dodecaboran-9-ylmethyl)-1,7-dicarba-*closo*-dodecaborane) (**SP2**), confirm these assumptions (more information is given in the electronic supporting information).

The introduction of the thiol group at position 9 of the central carborane cluster in **4** was carried out using the procedure for unsubstituted *ortho*- and *meta*-carboranes (Scheme 1, steps f and g) [70]. Reduction of the disulfide 1,2-bis[1,7-bis(1,7-dicarba-*closo*-dodecaboran-9-ylmethyl)-1,7-dicarba-*closo*-dodecaboran-9-yl]disulfane (**5**) was straightforward following the given procedure [70], but the work-up after reduction of **5** to 1,7-bis(1,7-dicarba-*closo*-dodecaboran-9-ylmethyl)-1,7-dicarba-*closo*-dodecaboran-9-yl-thiol (**6**) was slightly modified. The disulfide **5** and the thiol **6** were isolated in good to very good yield.

**Figure 2:**
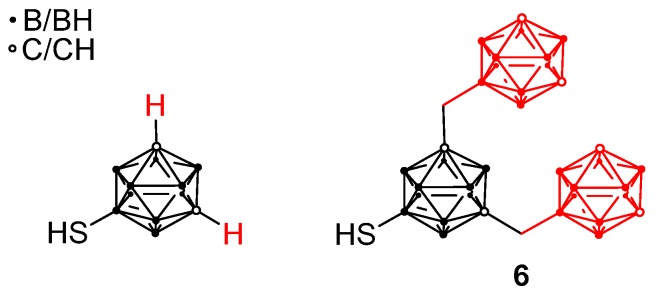
Comparison between 9-mercapto-1,7-dicarba-*closo*-dedecaboran(12) (left) and 1,7-bis(1,7-dicarba-*closo*-dodecaboran-9-ylmethyl)-1,7-dicarba-*closo*-dodecaboran-9-yl-thiol (**6**) (right) (altered fragments are drawn in red).

A synthetic protocol which was developed for 9-mercapto-1,7-dicarba-*closo*-dodecaborane(12) was adapted to the reaction of thiol **6** (Figure 2) to give the final product **7** with an increased boron load (Scheme 1, step h) [45,46,47].

The *s*-triazinyl dichloride **8** was prepared according to the procedure described in our recent work [46,47]. Product **7** was obtained in very good yield; the overall yield about seven steps, starting from *meta*-carborane, including the synthesis of compound **1**, is about 10%.

All synthesized compounds were fully characterized by NMR and IR spectroscopy, mass spectrometry, elemental analysis and melting point determination. For **4** and **6**, single crystals were obtained suitable for X-ray diffraction.

**Figure 3:**
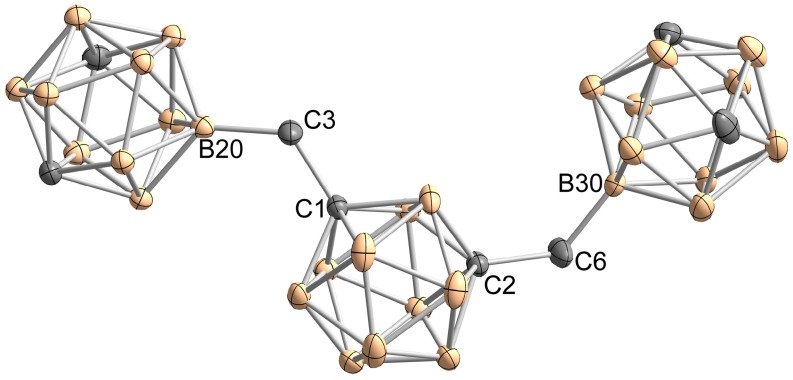
Molecular structure of 1,7-bis(1,7-dicarba-*closo*-dodecaboran-9-ylmethyl)-1,7-dicarba-*closo*-dodecaborane (**4**). Hydrogen atoms are omitted for clarity. Thermal ellipsoids are drawn at the 50% probability level. Selected bond lengths [pm] and bond angles [°]: C1–C3 153.5(2), C3–B20 160.1(3), C2–C6 153.0(2), C6–B30 159.6(3); C1–C3–B20 123.0(2), C2–C6–B30 124.2(2).

Although the appearance of compound **4** is quite simple, characterization was not trivial. Due to the large number of different boron atoms, the characterization of **4** via NMR was challenging. In the ^1^H NMR spectrum, there are three significant signals observable. First, at 1.38 ppm to 3.42 ppm the typical “multiplet” (very broad signal appearing as a multiplet formed by overlapping of all BH proton signals) of the BH groups is observed (28 protons). Second, two very intense and broad singlets, with an integral of four protons each, were observed at 1.89 ppm and 2.88 ppm. Considering the electron-withdrawing effect of the carborane moieties, the signal at 2.88 ppm was assigned to the cluster CH groups and the signal at 1.89 ppm to the methylene groups in **4**. This assumption was further corroborated by 2D-NMR (HSQC and HMBC) spectroscopy. In the ^13^C NMR spectrum of compound **4** using the pulse sequence for an “attached proton test” experiment (APT), three signals were observed. The signal at 25.9 ppm has a very broad appearance and can be assigned to the CH_2_ group; line broadening is caused by the connected boron atoms of the two terminal carborane clusters. The second signal at 54.0 ppm can easily be identified as the cluster CH group. These observations were also confirmed by 2D-NMR experiments. The third signal at 76.1 ppm is clearly identified by 2D-HMBC NMR as the quaternary carbon atoms in the central carborane cluster. The interpretation of the ^11^B NMR experiments was more difficult. Due to the magnetic inequivalence of the boron atoms in **4**, many boron signals were observed in the NMR spectrum. Only the signal at −1.9 ppm could be directly interpreted as the boron atom at position 9 in both terminal carborane clusters based on the proton-coupled ^11^B NMR spectrum, where this signal is still observed as a singlet. Single crystals of **4** were obtained from ethyl acetate/*n*-hexane solution. The molecular structure (Figure 3) confirms the spectroscopic assignments.

Like **4**, disulfide 1,2-bis[1,7-bis(1,7-dicarba-*closo*-dodecaboran-9-ylmethyl)-1,7-dicarba-*closo*-dodecaboran-9-yl]disulfane (**5**) shows the typical signals in the ^1^H NMR spectrum: BH protons in a range from 1.39 ppm to 3.57 ppm as a very broad multiplet, protons of the bridging methylene groups at 1.89 and 1.91 ppm, and the cluster CH groups at 2.89 ppm, all as broad singlets. All integrals in the ^1^H NMR spectrum correspond to the respective atom numbers in the sum formula of **5**. The ^11^B NMR spectrum of compound **5** is more surprising. Three singlets are observed, indicating the presence of three different B–S groups. Apparently, compound **5** is readily oxidized under ambient conditions, as mass spectrometry indicated the formation of the mono-oxidized derivative *S*-[1,7-bis(1,7-dicarba-*closo*-dodecaboran-9-ylmethyl)-1,7-dicarba-*closo*-dodecaboran-9-yl]-1,7-bis(1,7-dicarba-*closo*-dodecaboran-9-ylmethyl)-1,7-dicarba-*closo*-dodecaborane-9-sulfinthioate (**5′**) (see experimental section). Unsymmetrical oxidized disulfides are already known [71].

**Figure 4:**
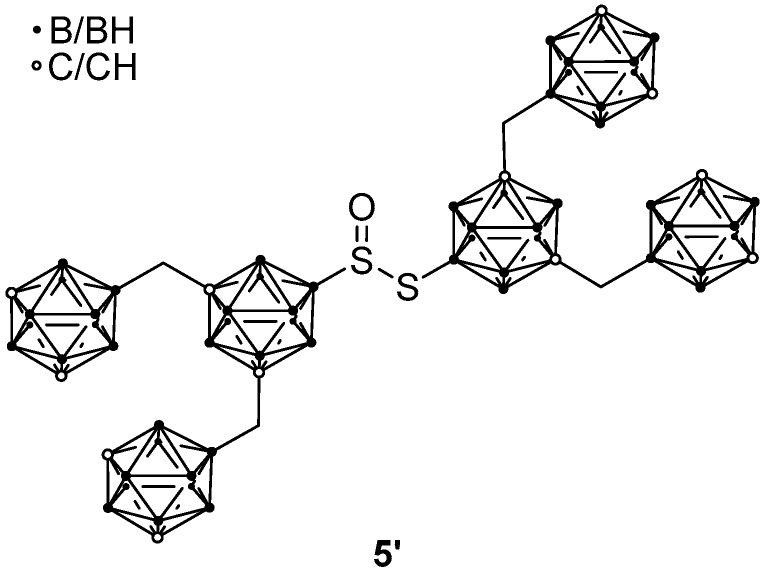
Mono-oxidized derivative of 1,2-bis[1,7-bis(1,7-dicarba-*closo*-dodecaboran-9-ylmethyl)-1,7-dicarba-*closo*-dodecaboran-9-yl]disulfane (**5′**).

This observation explains the presence of the three different B–S groups in the ^11^B NMR spectrum: The disulfide **5** exhibits one signal and the unsymmetrical thiosulfinate **5′** (Figure 4) two (1:1 mixture of **5** and **5′**). All other boron atoms in **5** and **5′** are observed as a mixture of overlapping multiplets and singlets, which do not allow further assignment. Fortunately, the presence of this oxidized species besides **5** is no problem for the synthesis of **6**, as the disulfide is cleaved under reductive conditions, which also reduce the oxidized species **5′**.

**Figure 5:**
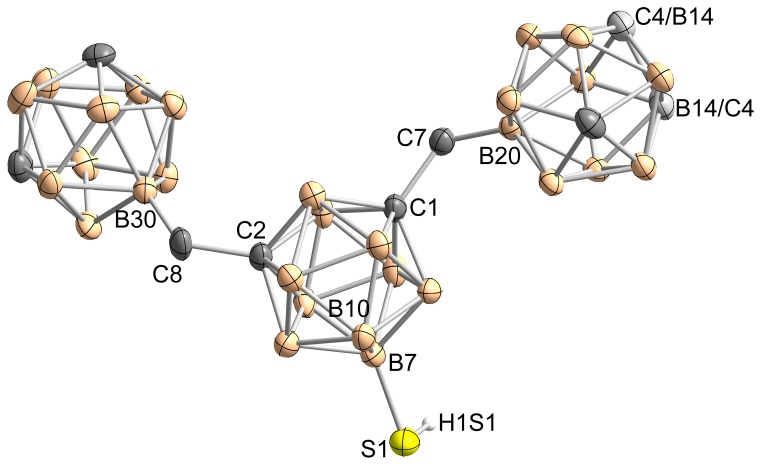
Molecular structure of 1,7-bis(1,7-dicarba-*closo*-dodecaboran-9-ylmethyl)-1,7-dicarba-*closo*-dodecaboran-9-ylthiol (**6**). Thermal ellipsoids are drawn at the 50% probability level. Carbon/boron disorder in one of the carborane clusters in a ratio of 0.55(2):0.45(2) (shown) and the central carborane unit (ratio of 0.938(1):0.062(1), not shown) is observed. Hydrogen atoms (other than SH) are omitted for clarity. Selected bond lengths [pm] and bond angles [°]: S1–B7 187.0(4), C1–C7 152.9(3), C7–B20 159.9(4), C2–C8 152.5(4), C8–B30 160.3(4); C1–C7–B20 122.7(2), C2–C8–B30 124.2(2).

As expected, the spectroscopic data of 1,7-bis(1,7-dicarba-*closo*-dodecaboran-9-ylmethyl)-1,7-dicarba-*closo*-dodecaboran-9-yl-thiol (**6**) are similar to **4**. In addition to the proton signals of the CH and BH groups of the carborane clusters and the CH_2_ signals for the bridging methylene groups, a multiplet at 0.39 ppm is observed for the thiol group in **6**, comparable with 9-mercapto-1,7-dicarba-*closo*-dodecaborane(12) [70]. The molecular structure of **6** is shown in Figure 5.

**Figure 6:**
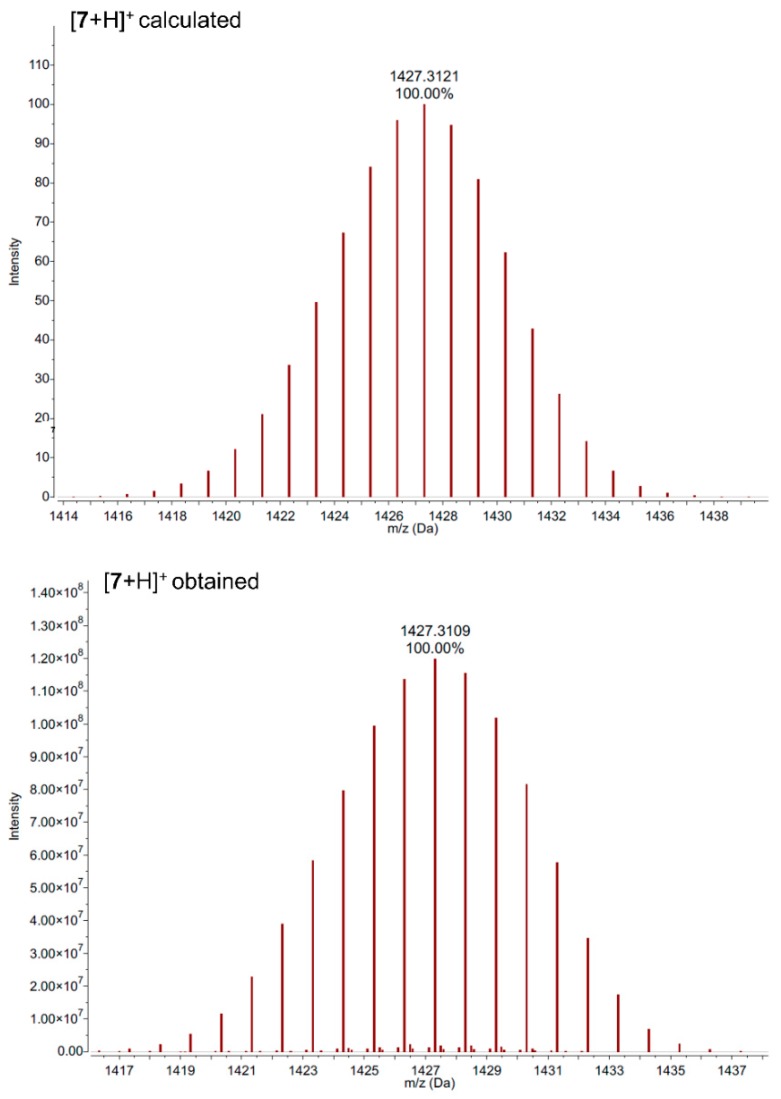
Comparison of the isotopic pattern of the calculated (top) and measured signal (bottom) of [**7** + H]^+^ [41].

Compound **7** showed the expected signals in the ^1^H NMR spectrum, including a broad multiplet from 1.5 to 3.5 ppm corresponding to 54 BH groups. Furthermore, all expected signals were observed in the ^13^C, ^11^B and ^11^B{^1^H} NMR spectra. High-resolution electrospray-ionization mass spectrometry showed the signal for protonated **7** ([**7** + H]^+^, calculated *m*/*z* 1427.3121, obtained *m*/*z* 1427.3109) (Figure 6) with an impressive isotopic pattern.

## 3. Materials and Methods

Materials and methods: All reactions were carried out under nitrogen atmosphere using Schlenk techniques, if not reported otherwise. Anhydrous diethyl ether and dichloromethane were obtained with an MBRAUN solvent purification system MB SPS-800. Acetonitrile, benzene and 2,4,6-collidine were dried over CaH_2_ and distilled. Anhydrous tetrahydrofuran was dried over potassium and distilled. All solvents were stored over molecular sieves (3 Å) under nitrogen atmosphere. 9-Iodo-1,7-dicarba-*closo*-dodecaborane(12) (**1**) [62,63,64], 1,7-bis(hydroxymethyl)-1,7-dicarba-*closo*-dodecaborane(12) (**2**) [66], 1,7-bis(bromomethyl)-1,7-dicarba-*closo*-dodecaborane(12) (**3**) [65], and *tert*-butyl-*N*-(4,6-dichloro-1,3,5-triazin-2-yl)-*N*-(1′,2′:3′,4′-di-*O*-isopropylidene-6′-deoxy-α-d-galactopyranos-6′-yl)glycinate (**8**) [46,47] were prepared according to the literature. All other chemicals were purchased and used as received.

**Figure 7:**
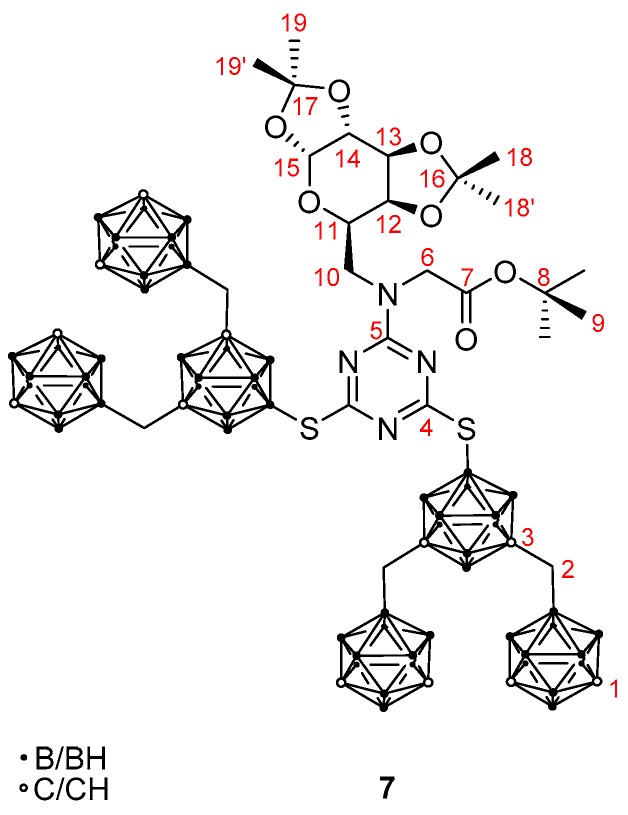
Numbering scheme of compound **7**.

Thin-layer chromatography (TLC) with silica gel 60 F_254_ on glass available from Merck KGaA was used for monitoring the reactions. Carborane-containing spots were visualized with a 5–10% solution of PdCl_2_ in methanol. For chromatography, silica gel (60 Å) with a particle diameter in the range of 0.035 to 0.070 mm, the Biotage^®^ Isolera 1 or the Biotage^®^ Isolera 4 automatic purification system with SNAP (particle diameter in the range of 0.040 to 0.065 mm) and SNAP Ultra (spherical particles, diameter 0.025 mm) cartridges were used. The triazine and carborane species were detected by an integrated UV/Vis detector (Isolera 1) or evaporative light scattering detector (ELSD) A-120 (Isolera 4). For chromatography, solvents were distilled before use. NMR measurements were carried out on a Bruker AVANCE III HD spectrometer with an Ascend™ 400 magnet at room temperature. Tetramethylsilane was used as internal standard for ^1^H and ^13^C{^1^H} NMR spectra, and ^11^B and ^11^B{^1^H} NMR spectra were referenced to the Ξ scale [72]. NMR spectra were recorded at the following frequencies: ^1^H—400.16 MHz, ^13^C—100.63 MHz, ^11^B—128.38 MHz. All chemical shifts are reported in parts per million (ppm). Assignment of the ^1^H and ^13^C signals was based on 2D NMR spectra (H,H-COSY, HSQC, HMQC, HMBC). Identification of the boron atom attached to sulfur was possible by comparison of the proton-coupled and -decoupled ^11^B NMR spectra. NMR data were interpreted with MestReNova [73]. NMR signals that appear as broad overlapping signals with the shape of a multiplet in either ^1^H, ^11^B{^1^H} or ^11^B NMR spectra are described as ‘br’ (broad). In this case, the superscript a is added (br^a^). The numbering scheme of compound **7** for assignments of NMR signals is given at the end of the experimental section (Figure 7). IR data were obtained with a PerkinElmer FT-IR spectrometer Spectrum 2000 as KBr pellets and on a Thermo Scientific Nicolet iS5 with an ATR unit in the range of 4000 to 400 cm^−1^. Electrospray ionization mass spectrometry was performed with an ESI ESQUIRE 3000 PLUS spectrometer with an IonTrap-analyzer from Bruker Daltonics or on a MicroTOF spectrometer from Bruker Daltonics with a ToF analyzer in negative or positive mode. Dichloromethane, acetonitrile, methanol, or mixtures of these solvents, were used as solvents for the measurements. Electron impact mass spectrometry was performed with a MAT 8230 spectrometer with a sector field analyzer from Thermo Scientific. Elemental analysis was conducted with a VARIO EL elemental analyzer from Heraeus. Melting points were determined with a Gallenkamp MPD350.BM2.5 melting point device and are not corrected.

X-ray diffraction experiments: Measurements were performed with a Gemini diffractometer (Rigaku Oxford Diffraction) with Mo-K_α_ radiation (λ = 71.073 pm), ω-scan rotation. Data reduction was performed with CrysAlis Pro [74], including the program SCALE3 ABSPACK [75] for empirical absorption correction. The structures were solved by dual space methods (SHELXT-2014) [76] and the refinement of all non-hydrogen atoms was performed with SHELXL-2018 [77]. For **6**, H atoms (except SH) were calculated on idealized positions. In all other cases, H atoms were located on difference Fourier maps calculated at the final stage of the structure refinement. Structure figures were generated with Diamond [78]. The electronic supporting information and CCDC 1945754 (**4**), 1945755 (**6**), 1945756 (**SP1**) and 1945757 (**SP2**) contain the supplementary crystallographic data for this paper. The crystallographic data can be obtained free of charge via www.ccdc.cam.ac.uk/conts/retrieving.html (or from the Cambridge Crystallographic Data Centre, 12 Union Road, Cambridge CB2 1EZ, UK; fax: (+44)1223-336-033; or deposit@ccdc.cam.uk).

*1,7-Bis(1,7-dicarba-closo-dodecaboran-9-ylmethyl)-1,7-dicarba-closo-dodecaborane* (**4**): A 250 mL two-necked round bottom flask equipped with a condenser and a dropping funnel was charged with 6.97 g (287 mmol, 30.1 eq.) Mg turnings, evacuated and purged with nitrogen. The Mg turnings were activated by mechanical stirring and suspended in 10 mL dry tetrahydrofuran. A second Schlenk flask was charged with 3.14 g (9.52 mmol, 1.00 eq.) 1,7-bis(bromomethyl)-1,7-dicarba-*closo*-dodecaborane(12) (**3**) and 80 mL dry tetrahydrofuran. The carborane solution was transferred into the dropping funnel and slowly added to the Mg turnings. The reaction mixture was stirred under reflux conditions for 2 h. A 250 mL two-necked round bottom flask with a condenser and a dropping funnel was evacuated and purged with nitrogen, and 3.00 g (17.6 mmol, 1.85 eq.) 9-iodo-1,7-dicarba-*closo*-dodecaborane(12) (**1**) were added and dissolved in 60 mL dry tetrahydrofuran. The mixture was cooled to 0 °C. The Grignard reagent from the first reaction step was filtered, transferred into the second dropping funnel and slowly added to 9-iodo-*m*-carborane (**1**) at 0 °C. The mixture was stirred for 30 min at room temperature. Subsequently, 0.34 g (1.79 mmol, 0.19 eq.) copper(I) iodide and 0.78 g (1.11 mmol, 0.12 eq.) bis(triphenylphosphine)palladium(II) dichloride were added and the mixture was stirred under reflux for 2 d. The reaction was stopped by adding 30 mL saturated NaCl solution and 15 mL 2 M hydrochloric acid. The aqueous layer was separated from the organic one and extracted three times each with 30 mL ethyl acetate. The combined organic layers were dried over MgSO_4_, filtered and the solvent was removed under reduced pressure. The raw product was purified by column chromatography (*n*-hexane/ethyl acetate, 10:1, *v*/*v*) and 1.32 g (2.89 mmol, 30%, R*_f_* = 0.42) of compound **4** were isolated as a colorless solid. 1-Methyl-7-(1,7-dicarba-*closo*-dodecaboran-9-ylmethyl)-1,7-dicarba-*closo*-dodecaborane (**SP2**) was obtained in 18% yield (529 mg, 1.68 mmol, R*_f_* = 0.58, 10:1, *n*-hexane/ethyl acetate, *v*/*v*) and 1-(hydroxymethyl)-7-(1,7-dicarba-*closo*-dodecaboran-9-ylmethyl)-1,7-dicarba-*closo*-dodecaborane (**SP1**) was obtained in 8% yield (252 mg, 762 µmol, R*_f_* = 0.31, 10:1, *n*-hexane/ethyl acetate, *v*/*v*) (additional analytical data are given in the supplementary information). Colorless crystals of **4** suitable for X-ray structure determination were obtained from CHCl_3_ at room temperature. Crystallographic data are given in Table S4, and the molecular structure is depicted in Figure 3. T_m_: 182–185 °C (acetone). IR (KBr): ṽ = 3420 (w, H bridges), 3060 (s, νC_sp2_H), 2926 (w, νC_sp3_H), 2906 (w, νC_sp3_H), 2601 (s, νBH), 1733 (w), 1418 (m, δCH_2_), 1291 (w), 1249 (w), 1184 (w), 1159 (m), 1105 (w), 1068 (m), 1009 (m), 980 (m), 923 (w), 851 (w), 831 (w), 811 (w), 724 (m, CH_2_ rocking), 683 (w), 538 (w), 519 (w) cm^−1^. ^1^H NMR (CDCl_3_): δ = 1.38–3.42 (m, vbr, 28 H, 2xB_10_H_9_, 1xB_10_H_10_), 1.89 (s, br, 4 H, 2xCH_2_), 2.88 ppm (s, br, 4 H, 4xCH_Cluster_). ^13^C{^1^H} NMR (CDCl_3_): δ = 25.9 (s, vbr, CH_2_, 2xCH_2_), 54.0 (s, br, CH, 4xCH_Cluster_), 76.1 ppm (s, br, C_q_, C_q,Cluster_). ^11^B{^1^H} NMR (CDCl_3_): δ = −20.3 to −16.3 (m, br, 4 B), −14.1 (s, 4 B), −13.1 (s, 4 B), −12.5 to −8.8 (m, br, 10 B), −6.1 (m, 6 B), −1.9 ppm (s, 2 B, BC). ^11^B NMR (CDCl_3_): δ = −21.0 to −16.3 (m, br, 4 B), −15.6 to −8.6 (m, br, 18 B), −8.1 to −4.3 (m, 6 B), −1.9 ppm (s, 2 B, BC). LRMS (EI): C_8_H_36_B_30_, *m*/*z* calcd: 456.6 ([M]^+^); found: 456.6.

*1,2-Bis[1,7-bis(1,7-dicarba-closo-dodecaboran-9-ylmethyl)-1,7-dicarba-closo-dodecaboran-9-yl]disulfane* (**5**): A 100 mL two-necked round bottom flask, equipped with a condenser, was charged with 0.51 g (1.12 mmol, 1.00 eq.) 1,7-bis(1,7-dicarba-*closo*-dodecaboran-9-ylmethyl)-1,7-dicarba-*closo*-dodecaborane (**4**) and 0.19 g (1.42 mmol, 1.27 eq.) anhydrous AlCl_3_. The starting materials were suspended in 50 mL anhydrous CH_2_Cl_2_ and cooled to 0 °C. 0.05 mL (0.08 g, 0.63 mmol, 0.56 eq.) S_2_Cl_2_ dissolved in 2 mL anhydrous CH_2_Cl_2_ were added slowly to this mixture. The reaction mixture was stirred for 4 h under reflux. The reaction was stopped by pouring the mixture onto crushed ice. The resulting aqueous layer was extracted three times each with 30 mL CH_2_Cl_2_. The combined organic layers were washed once with 30 mL distilled H_2_O, dried over MgSO_4_, filtered off and the solvent was removed under reduced pressure. The raw product was purified by column chromatography using the Isolera Four device (SNAP Ultra 25 g cartridge, 12 mL/min, *n*-hexane/ethyl acetate 49:1 to 41:9, *v*/*v*). 0.41 g (0.42 mmol, 67%) of compound **5** were isolated as an off-white crystalline solid. During characterization of **5**, partial oxidization occurred. T_m_: 109–112 °C (ethyl acetate). IR (KBr): ṽ = 3432 (w, H bridges), 3059 (m, νC_sp2_H), 2925 (m, νC_sp3_H), 2907 (m, νC_sp3_H), 2597 (s, νBH), 1710 (w), 1684 (m), 1616 (m), 1443 (w, δCH_2_), 1419 (m, δCH_2_), 1378 (w), 1356 (w), 1288 (w), 1220 (w), 1159 (m), 1106 (w), 1068 (m), 1012 (s, νBS), 980 (m), 960 (m), 923 (w), 897 (w), 847 (m), 723 (s, CH_2_ rocking), 620 (w), 587 (w), 534 (w), 517 (w), 485 (w) cm^−1^. ^1^H NMR (CDCl_3_): δ = 1.39–3.57 (m, vbr, 54 H, 6xB_10_H_9_), 1.89, 1.91 (s, br, 8 H, 4xCH_2_), 2.89 ppm (s, br, 8 H, 8xCH_Cluster_). ^13^C{^1^H} NMR (CDCl_3_): δ = 26.3 (s, vbr, CH_2_, 4xCH_2_), 54.1 (s, br, CH, 8xCH_Cluster_), 74.9, 75.4, 75.6 ppm (s, C_q_, 4xC_q,Cluster_). ^11^B{^1^H} NMR (CDCl_3_): δ = −19.3 (s, br, 4 B), −17.5 (s, 4 B), −14.0 (s, 8 B), −13.1 (s, 8 B), −10.8 (s, br, 6 B), −9.7 (s, br, 12 B), −6.1 (s, br, 12 B), −2.1 (s, 4 B, BC), −0.4, 0.2, 0.5 ppm (s, BS/BS and BS/BS(O)). ^11^B NMR (CDCl_3_): δ = −18.5 (m, br, 8 B), −13.5 (m, 16 B), −10.4 (m, br, 18 B), −6.1 (d, br, ^1^*J*_BH_ = 163 Hz, 12 B), −2.1 (s, 4 B, BC), −0.4, 0.2, 0.5 ppm (s, BS/BS and BS/BS(O)). HRMS (ESI+): C_16_H_70_B_60_OS_2_, *m*/*z* calcd: 993.0939 ([M_ox._ + H]^+^); found: 993.0937; (ESI−): C_16_H_70_B_60_S_2_, *m*/*z* calcd: 1011.0619 ([M + Cl]^−^); found: 1011.0673. ^11^B NMR spectroscopy and mass spectrometry indicate the presence of the oxidized derivative **5′**. Fortunately, the presence of this oxidized species besides **5** is no problem for the synthesis of **6**, as the disulfane is cleaved under reductive conditions, which also reduce the oxidized species **5′**.

*1,7-Bis(1,7-dicarba-closo-dodecaboran-9-ylmethyl)-1,7-dicarba-closo-dodecaboran-9-yl-thiol* (**6**): A 100 mL round bottom flask, equipped with a condenser, was charged with 0.20 g (0.21 mmol, 1.00 eq.) 1,2-bis[1,7-bis(1,7-dicarba-*closo*-dodecaboran-9-ylmethyl)-1,7-dicarba-*closo*-dodecaboran-9-yl]disulfane (**5**) and 20 mL of a 1:1 mixture of concentrated hydrochloric acid and glacial acetic acid, as well as 10 mL ethyl acetate were added for a better solubility. The mixture was heated to reflux for 4 h and excess of zinc powder (1.10 g, 16.8 mmol, 80.0 eq.) was added in 12 portions. The mixture was cooled to room temperature and the acid neutralized with Na_2_CO_3_ and NaHCO_3_. The resulting aqueous layer was extracted three times each with 30 mL CH_2_Cl_2_. The combined organic layers were washed once with distilled H_2_O, dried over MgSO_4_, filtered and the solvent was removed under reduced pressure. The raw product was purified by column chromatography (*n*-hexane/ethyl acetate, 7:1 to 3:1, *v*/*v*) and 83 mg (0.17 mmol, 81%) of compound **6** were isolated as an off-white solid. Colorless crystals of **6** suitable for X-ray structure determination were obtained from CHCl_3_ at room temperature. Crystallographic data are given in Table S4, and the molecular structure is depicted in Figure 5. T_m_: 180–183 °C (toluene). IR (KBr): ṽ = 3416 (w, H bridges), 3055 (m, νC_sp2_H), 3031 (s, νC_sp2_H), 2923 (m, νC_sp3_H), 2907 (m, νC_sp3_H), 2850 (m, νSH), 2599 (s, νBH), 1466 (w, δCH_2_), 1418 (m, δCH_2_), 1289 (w), 1159 (m), 1108 (w), 1068 (m), 1011 (s, νBS), 980 (m), 962 (m), 924 (w), 893 (w), 851 (m), 776 (w), 759 (w), 722 (m), 689 (w), 664 (w), 588 (w), 517 (w), 437 (w) cm^−1^. ^1^H NMR (CDCl_3_): δ = 0.39 (m, 1 H, SH), 1.39–3.75 (m, vbr, 27 H, 3xB_10_H_9_), 1.89 (s, br, 4 H, 2xCH_2_), 2.89 ppm (s, br, 4 H, 4xCH_Cluster_). ^13^C{^1^H} NMR (CDCl_3_): δ = 26.0 (s, vbr, CH_2_, 2xCH_2_), 53.3, 55.1 (s, br, CH, 4xCH_Cluster_), 76.0 ppm (s, br, C_q_, 2xC_q,Cluster_). ^11^B{^1^H} NMR (CDCl_3_): δ = −19.3 (s, br, 2 B), −17.6 (s, 2 B), −14.0 (s, 4 B), −13.2 (s, 4 B), −11.0 (s, br, 3 B), −9.8 (s, br, 6 B), −6.1 (s, br, 6 B), −3.4 (s, 1 B, BS), −2.1 ppm (s, 2 B, BC). ^11^B NMR (CDCl_3_): δ = −18.7 (m, br, 4 B), −13.5 (m, 8 B), −10.4 (m, br, 9 B), −6.1 (d, br, ^1^*J*_BH_ = 162 Hz, 6 B), −3.4 (s, 1 B, BS), −2.1 ppm (s, 2 B, BC). HRMS (ESI−): C_8_H_36_B_30_S, *m*/*z* calcd: 487.5469 ([M − H]^−^); found: 487.5438.

*tert-Butyl-*N*-{4,6-bis[(1,7-dicarba-*closo*-dodecaboran-9-ylmethyl)1,7-dicarba-*closo*-dodecaboran-9-ylthio]-1,3,5-triazin-2-yl}-*N*-(1′,2′:3′,4′-di-*O*-isopropylidene-6′-deoxy-α-d-galactopyranos-6′-yl)glycinate* (**7**): A 100 mL round bottom flask, equipped with a condenser, was charged with 97 mg (0.19 µmol, 1.00 eq.) *tert*-butyl-*N*-(4,6-dichloro-1,3,5-triazin-2-yl)-*N*-(1′,2′:3′,4′-di-*O*-isopropylidene-6′-deoxy-α-d-galactopyranos-6′-yl)glycinate (**8**), 290 mg (0.59 µmol, 3.12 eq.) 1,7-bis(1,7-dicarba-*closo*-dodecaboran-9-ylmethyl)-1,7-dicarba-*closo*-dodecaboran-9-ylthiol (**6**) and 133 mg (0.96 µmol, 5.06 eq.) K_2_CO_3_. This mixture was suspended in 30 mL dry MeCN and was heated to reflux for 2 d. The mixture was cooled to room temperature and 15 mL saturated NaCl solution were added. The aqueous layer was extracted three times each with 10 mL ethyl acetate. The combined organic layers were dried over MgSO_4_, filtered and the solvent was removed under reduced pressure. The raw product was purified by column chromatography using the Isolera One device with a 25 g SNAP Ultra cartridge (*n*-hexane/ethyl acetate, 47:3 to 1:1, *v*/*v*, 20 mL/min) to obtain 216 mg (15.1 µmol, 79%, R*_f_* = 0.18, *n*-hexane/ethyl acetate, 1:3, *v*/*v*) of **7** as an off-white solid. T_m_: 176–178 °C (methanol/water). IR (KBr): ṽ = 3436 (w, H bridges), 3061 (m, νC_sp2_H), 2978 (m, νC_sp3_H), 2929 (m, νC_sp3_H), 2909 (m, νC_sp3_H), 2600 (s, νBH), 1740 (m, νC=O), 1640 (w), 1530 (s, νC=N), 1511 (s, νC=N), 1481 (s, δCH_2_), 1422 (w), 1371 (w), 1316 (w), 1255 (m), 1219 (m), 1162 (s, νCO), 1112 (w), 1070 (s, νCO), 1011 (s, νBS), 980 (m), 921 (w), 903 (w), 881 (w), 846 (s), 803 (w), 725 (m), 696 (w), 590 (w), 513 (w) cm^−1^. ^1^H NMR (CDCl_3_): δ = 1.28 (s, 3H, C^18 or 18′^H_3_), 1.36 (s, 3H, C^19 or 19′^H_3_), 1.40 (s, 3 H, C^18 or 18′^H_3_), 1.42 (s, 3 H, C^19 or 19′^H_3_), 1.48 (s, 9 H, C(C^9^H_3_)_3_), 1.54–3.46 (br^a^, 54 H, 6xB_10_H_9_), 1.95 (s, br, 8 H, 4xC^2^H_2_), 3.59 (s, br, 8 H, 8xC^1^H), 4.14 (dd, ^2^*J*_HH_ = 14.5 Hz, ^3^*J*_HH_ = 3.4 Hz, 1 H, C^10^*H*H), 4.22 (m, 1 H, C^10^H*H*), 4.35 (m, 4 H, C^11^H, C^13^H, C^14^H, C^6^*H*H), 4.62 (dd, ^3^*J*_HH_ = 7.9 Hz, ^3^*J*_HH_ = 2.4 Hz, 1 H, C^12^H), 4.88 (d, ^2^*J*_HH_ = 17.7 Hz, 1 H, C^6^H*H*), 5.48 ppm (d, ^3^*J*_HH_ = 4.9 Hz, 1 H, C^15^H). ^13^C{^1^H} NMR (CDCl_3_): δ = 24.7, 25.2, 26.3 and 26.4 (s, CH_3_, C^18^H_3_, C^18′^H_3_, C^19^H_3_ and C^19′^H_3_), 27.2 (s, br, CH_2_, C^2^H_2_), 28.4 (s, CH_3_, C(C^9^H_3_)_3_), 48.4 (s, CH_2_, C^10^H_2_), 50.7 (s, CH_2_, C^6^H_2_), 55.8 (s, CH, 8xC^1^H), 67.2 (s, CH, C^11^H), 71.2 (s, CH, C^14^H), 71.7 (s, CH, C^12^H), 72.4 (s, CH, C^13^H), 75.97, 76.05, 76.11 (s, C_q_, 4xC_q_^3^), 81.6 (s, C_q_, C_q_^8^), 97.1 (s, CH, C^15^H), 109.2 (s, C_q_, C_q_^17^), 109.7 (s, C_q_, C_q_^16^), 164.1 (s, C_q_, C_q_^5^), 169.4 (s, C_q_, C_q_^4^), 179.2 ppm (s, C_q_, C_q_^7^). ^11^B{^1^H} NMR (CDCl_3_): δ = −19.0 (s, br, 4 B), −17.3 (s, 4 B), −14.0 (s, 16 B), −13.0 (s, br, 6 B), −10.0 (s, 12 B), −6.3 (s, br, 12 B), −4.2 (s, 2 B, BS), −2.3 ppm (s, br, 4 B, BC). ^11^B NMR (CDCl_3_): δ = −18.1 (m, br, 8 B), −13.4 (m, br, 22 B), −10.0 (d, ^1^*J*_BH_ = 147 Hz, 12 B), −6.3 (d, ^1^*J*_BH_ = 156 Hz, 12 B), −4.2 (s, 2 B, BS), −2.3 ppm (s, 4 B, BC). HRMS (ESI+): C_37_H_100_B_60_N_4_O_7_S_2_, *m*/*z* calcd: 1427.3121 ([M + H]^+^); found: 1427.3109.

## 4. Conclusions

An unusual Kumada-like cross-coupling reaction between three carboranyl-substituted components was employed for the synthesis of a tris-*meta*-carborane derivative (**4**), which could be converted to the corresponding tris-*meta*-carboranyl thiol **6** and successively coupled with *tert*-butyl-*N*-(4,6-dichloro-1,3,5-triazin-2-yl)-*N*-(1′,2′:3′,4′-di-*O*-isopropylidene-6′-deoxy-α-d-galactopyranos-6′-yl)glycinate (**8**) using a modular approach, which was previously successfully employed for the smaller analog, 9-mercapto-1,7-dicarba-*closo*-dedecaboran(12) [46,47], to give the boron-rich compound **7**. Thus, **6** represents a highly facile boron-rich building block for the development of BNCT agents. Compound **7** contains 60 boron atoms, equivalent to a relative boron load of about 45%. After deprotection of the *tert*-butyl ester group, the resulting carboxylic acid will be suitable for coupling reactions with tumor-selective biomolecules.

## Supplementary Materials

The following are available online: Additional synthetic procedures and analytical data for compounds **1**, **2**, **3**, **SP1** and **SP2**; and full crystallographic data for compounds **4**, **6**, **SP1** and **SP2** are given in the electronic supplementary information.
