# Supplementary material for: Enlargement of a Modular System—Synthesis and Characterization of an s-Triazine-Based Carboxylic Acid Ester Bearing a Galactopyranosyl Moiety and an Enormous Boron Load
Source: Molecules. 2019 Sep 10;24(18):3288. doi: 10.3390/molecules24183288 (PMC6767515; doi:10.3390/molecules24183288)
Supplement: Supplementary file 1 [file molecules-24-03288-s001.pdf]

Electronic Supplementary Information

Enlargement of a Modular System – Synthesis and Characterization of an *s*-Triazine-based Carboxylic Acid Ester Bearing a Galactopyranosyl Moiety and an Enormous Boron Load

Martin Kellert,[a] Peter Lönnecke,[a] Bernd Riedl,[b] Johannes Koebberling,[b] and Evamarie Hey-Hawkins*[a]

[a] *Leipzig University, Faculty of Chemistry and Mineralogy, Institute of Inorganic Chemistry, Johannisallee 29, 04103 Leipzig, Germany*

[b] *Bayer AG, Aprather Weg 18A, 42113 Wuppertal, Germany*

* *Corresponding author. Phone: 0049-341-9736151, Fax: 0049-341-9739319, E-mail: hey@uni-leipzig.de.*

Table of Contents

1. Additional Analytical Data of **1**, **2** and **3** 3

2. 1-(Hydroxymethyl)-7-(1,7-dicarba-*closo*-dodecaboran-9-ylmethyl)-1,7-dicaraba-*closo*-dodecaborane (**SP1**) and 1-methyl-7-(1,7-dicarba-*closo*-dodecaboran-9-ylmethyl)-1,7-dicarba-*closo*-dodecaborane) (**SP2**) 4

3. Additional Analytical Data of **SP1** and **SP2** 7

4. Crystallographic Data of **4**, **6**, **SP1** and **SP2** 9

5. References 11

1. Additional Analytical Data of 1, 2 and 3

9-Iodo-1,7-dicarba-*closo*-dodecaborane(12) (1) [1,2]: Elemental analysis: $C_2H_{11}B_{10}I$, calcd (%): C 8.89, H 4.10; found (%): C 8.78, H 3.91. T_m: 113–115°C (*n*-hexane). IR (KBr): ṽ = 3389 (w, H bridges), 3047 (s, $vC_{sp2}H$), 2650 (m, vBH), 2611 (s, vBH), 2573 (s, vBH), 2087 (w), 1157 (s), 1105 (w), 1050 (m), 1023 (s), 986 (s), 936 (s), 914 (s), 845 (m), 829 (m), 752 (s), 725 (s), 674 (w), 614 (m), 573 (w) cm^{-1}. ^1H NMR (acetone-d_6): δ = 1.46 – 3.53 (m, vbr, 9 H, $B_{10}H_9$), 3.89 ppm (s, br, 2 H, $2xCH_{Cluster}$). $^{11}B\{^1H\}$ NMR (acetone-d_6): δ = –23.6 (s, br, 1 B, BI), –18.6 (s, 1 B), –16.5 (s, 1 B), –13.0 (s, 2 B), –11.8 (s, 2 B), –8.6 (s, 1 B), –5.7 ppm (s, 2 B). ^{11}B NMR (acetone-d_6): δ = –23.6 (s, br, 1 B, BI), –18.6 (d, $^1J_{BH}$ = 183 Hz, 1 B), –16.5 (d, $^1J_{BH}$ = 183 Hz, 1 B), –12.4 (m, 4 B), –8.6 (d, $^1J_{BH}$ = 154 Hz, 1 B), –5.7 ppm (d, $^1J_{BH}$ = 165 Hz, 2 B). HRMS (ESI–): $C_2H_{11}B_{10}I$, m/z calcd: 269.0831 ($[M–H]^–$); found: 269.0848.

1,7-Bis(hydroxymethyl)-1,7-dicarba-*closo*-dodecaborane(12) (2) [3]: Elemental analysis: $C_4H_{16}B_{10}O_2$, calcd (%): C 23.52, H 7.90; found (%): C 24.15, H 7.97. T_m: 185–188°C (acetone). IR (KBr): ṽ = 3286(s, H bridges), 2975 (w, $vC_{sp3}H$), 2944 (w, $vC_{sp3}H$), 2887 (w), 2731 (w), 2637 (s, vBH), 2596 (s, vBH), 2110 (w), 1632 (w), 1463 (m), 1375 (m), 1266 (m), 1179 (s, vCO), 1079 (s, vCO), 1051 (m), 1036 (m), 1001 (m), 943 (w), 912 (w), 820 (w), 782 (w), 732 (m), 705 (m), 657 (m), 633 (m), 578 (w), 557 (w), 503 (w), 484 (w) cm^{-1}. ^1H NMR ($CDCl_3$): δ = 1.43 – 3.59 (m, vbr, 10 H, $B_{10}H_{10}$), 1.84 (t, $^3J_{HH}$ = 7.3 Hz, 2 H, 2xOH), 3.82 ppm (d, $^3J_{HH}$ = 7.2Hz, 4 H, $2xCH_2$). $^{11}B\{^1H\}$ NMR ($CDCl_3$): δ = –14.9 (s, 2 B), –12.0 (s, 4 B), –10.9 (s, 2 B), –7.1 ppm (s, 2 B). HRMS (ESI–): $C_4H_{16}B_{10}O_2$, m/z calcd: 203.2075 ($[M–H]^–$); found: 203.2067.

1,7-Bis(bromomethyl)-1,7-dicarba-*closo*-dodecaborane(12) (3) [4]: ^1H NMR ($CDCl_3$): δ = 1.45 – 3.90 (m, vbr, 10 H, $B_{10}H_{10}$), 3.66 ppm (s, br, 4 H, $2xCH_2$). $^{13}C\{^1H\}$ NMR ($CDCl_3$): δ = 32.0 (s, CH_2, CH_2Br), 73.3 ppm (s, br, C_q, $C_{q,Cluster}$). $^{11}B\{^1H\}$ NMR ($CDCl_3$): δ = –13.1 (s, 2 B), –10.6 (s, br, 6 B), –5.9 ppm (s, br, 2 B). LRMS (ESI–): $C_4H_{14}B_{10}Br_2$, m/z calcd: 375.0 ($[M+HCO_2^–]^–$); found: 375.0.

2. 1-(Hydroxymethyl)-7-(1,7-dicarba-*closo*-dodecaboran-9-ylmethyl)-1,7-dicaraba-*closo*-dodecaborane (SP1) and 1-methyl-7-(1,7-dicarba-*closo*-dodecaboran-9-ylmethyl)-1,7-dicarba-*closo*-dodecaborane) (SP2)

SP1 and **SP2** are side products of the Kumada-like C–B cross coupling reaction between 1,7-bis(bromomethyl)-1,7-dicarba-*closo*-dodecaborane(12) (**3**) and 9-iodo-1,7-dicarba-*closo*-dodecaborane(12) (**1**) to prepare 1,7-bis(1,7-dicarba-*closo*-dodecaboran-9-ylmethyl)-1,7-dicarba-*closo*-dodecaborane (**4**) (Scheme S2). **SP1** and **SP2** were obtained in 8 and 18 % yield, respectively.

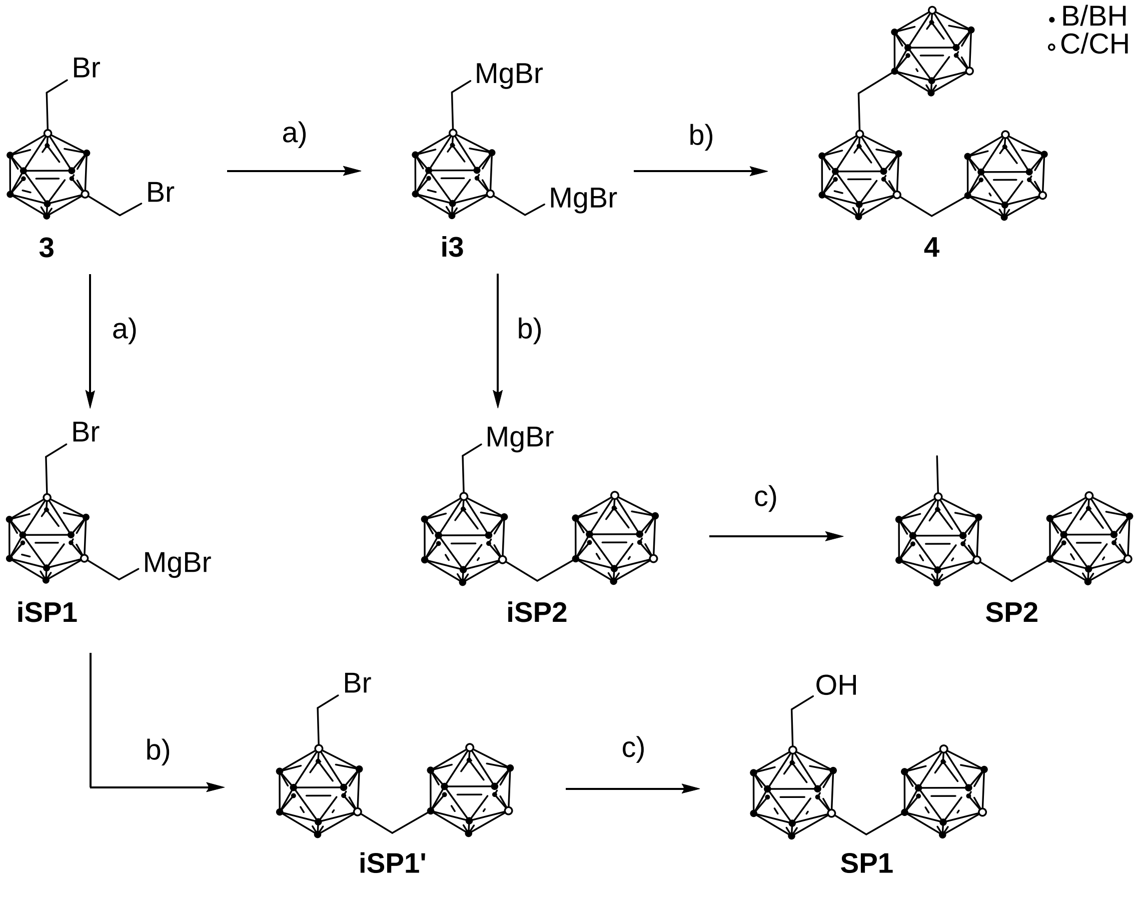

Scheme S1. Synthetic pathways to the formation of the side products **SP1** and **SP2**. a) Mg, tetrahydrofuran (THF), reflux, 2 h; b) **1**, CuI, [$PdCl_2(PPh_3)_2$], THF, reflux, 2 d; c) HCl, water.

The formation of **SP1** and **SP2** is the result of an incomplete conversion of the Grignard species (**iSP1**) or an incomplete C–B cross coupling reaction (**iSP2**) with following hydrolysis of the respective intermediates (**iSP1’** and **iSP2**) with hydrochloric acid. Both side products were characterized with the common analytical methods, and for both compounds, molecular structures were obtained. Compound **SP1** crystallized

from a chloroform/*n*-hexane solution with two molecules in the asymmetric unit (Figure S1) and forms hydrogen bonds between the two independent molecules in the asymmetric unit. Hydrogen bonding with two additional molecules of **SP1** result in formation of a square. Compound **SP2** crystallized from chloroform solution with one molecule in the asymmetric unit (Figure S2).

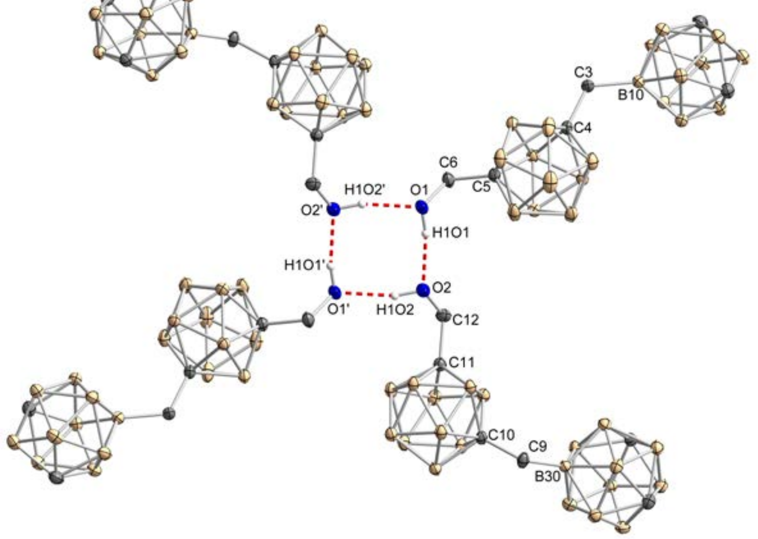

Figure S1. Molecular structure of **SP1**. Thermal ellipsoids are drawn at the 50% probability level. Hydrogen atoms at O1, O2, O1' and O2' are drawn with a fixed atom radius of 13.5 pm. All other H atoms are omitted for clarity. Selected bond lengths, distances [pm] and angles [°]: C5–C6 153.1(1), C6–O1 141.2(1), C4–C3 153.0(2), C3–B10 159.8(2), C11–C12 153.1(1), C12–O2 140.9(1), C10–C9 153.1(2), C9–B30 160.9(2), O1–H1O1 88(2), H1O1···O2 184(2), O1···O2 270.6(1), O2–H1O2 89(2), H1O2···O1' 187(2), O2···O1' 273.0(1); C4–C3–B10 123.3(1), C5–C6–O1 112.5(5), C10–C9–B30 122.7(1), C11–C12–O2 113.0(1), O1H1O1···O2 169(2), O2–H1O2···O1' 162(2).

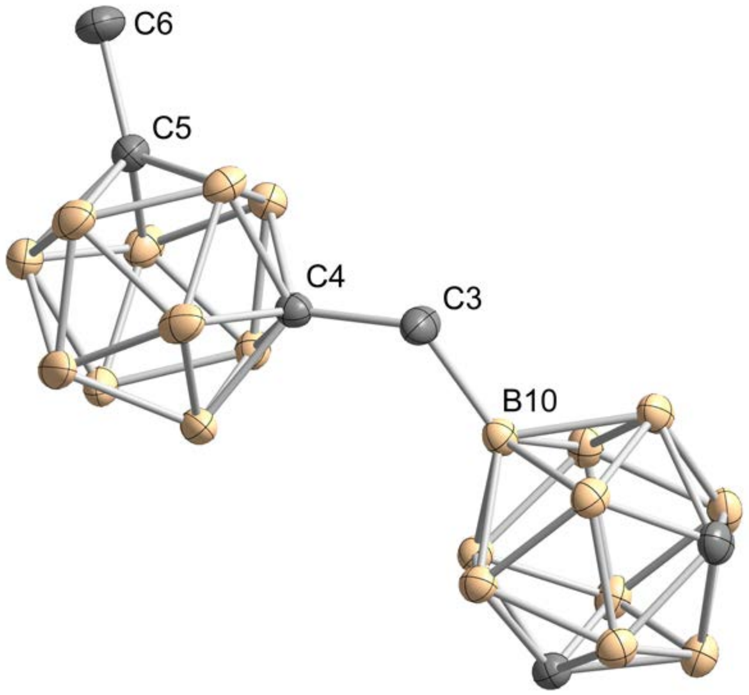

Figure S2. Molecular structure of **SP2**. Thermal ellipsoids are drawn at the 50% probability level. Hydrogen atoms are omitted for clarity. Selected bond lengths [pm] and bond angles [°]: C4–C3 153.8(2), C3–B10 160.3(3), C5–C6 151.9(3); C4–C3–B10 122.8(2).

Table S1. Comparison of selected bond lengths and bond angles in **SP1**, **SP2** and **4**. Corresponding C–C and C–B bond lengths and the C–C–B bond angles involve the methylene groups between two carborane clusters. Bond lengths and bond angles of the second molecule of **SP1** are given in brackets [].

	**SP1**	**SP2**	**4**
**C–C [pm]**	153.0(1) [153.1(1)]	153.8(2)[a]	153.0(2) – 153.5(2)
**C–B [pm]**	159.8(1) [160.9(1)]	160.3(3)	159.6(3) – 160.1(3)
**C–C–B [°]**	123.3(1) [122.7(1)]	122.9(1)	123.0(1) – 124.2(1)

[a]: The C–C bond length of the terminal methyl group in **SP2** is 151.9(3) pm.

Considering the selected C–C, C–B and C–C–B bond lengths and angles in **SP1**, **SP2** and **4**, no significant influence of the different substitution patterns is observed.

Although compounds **SP1** and **SP2** were isolated as side products, they are versatile starting materials and can be further modified. As an example, it is possible to convert the hydroxyl group in **SP1** into a leaving group for additional substitution reactions or to convert one or both residual CH groups in **SP2** to nucleophiles by reaction with organolithium reagents like *n*-butyllithium.

3. Additional Analytical Data of SP1 and SP2

1-(Hydroxymethyl)-7-(1,7-dicarba-*closo*-dodecaboran-9-ylmethyl)-1,7-dicaraba-*closo*-dodecaborane (SP1): This compound was isolated as a side product in the synthesis of compound **4** as a colorless crystalline solid (252 mg, 762 µmol, 8%, R_f = 0.31, 10:1, *n*-hexane/ethyl acetate, v/v; see synthetic procedure of **4**, main text). Colorless crystals of **SP1** suitable for X-ray structure determination were obtained from $CHCl_3$/*n*-hexane mixture at room temperature. Crystallographic data are given in Table S3, and the molecular structure is depicted in Figure S1. Elemental analysis: $C_6H_{26}B_{20}O$, calcd (%): C 21.81, H 7.93; found (%): C 21.89, H 8.17. T_m: 102–104°C (ethyl acetate). IR (KBr): ṽ = 3316 (m, H bridges), 3062 (m, $\nu C_{sp2}H$), 2931 (w, $\nu C_{sp3}H$), 2906 (w, $\nu C_{sp3}H$), 2601 (s, νBH), 1642 (w), 1459 (w), 1419 (m), 1380 (w), 1261 (w), 1174 (m), 1069 (s, νCO), 1012 (s, νCO), 980 (m), 923 (w), 857 (w), 836 (w), 811 (w), 723 (m), 667 (w), 516 (w) cm^{-1}. ^{1}H NMR ($CDCl_3$): δ = 1.33 – 3.65 (m, vbr, 19 H, $B_{10}H_9$ and $B_{10}H_{10}$), 1.90 (s, br, 2 H, CH_2), 2.91 (s, br, 2 H, $2xCH_{Cluster}$), 3.76 ppm (s, 2 H, *CH_2*OH). ^{13}C{^{1}H} NMR ($CDCl_3$): δ = 26.0 (s, br, CH_2, CH_2), 54.1 (s, br, CH, $2xCH_{Cluster}$), 65.2 (s, CH_2, *CH_2*OH), 76.7 (s, br, C_q, C_qCH_2), 76.8 ppm (s, br, C_q, C_qCH_2OH). ^{11}B{^{1}H} NMR ($CDCl_3$): δ = –19.3 (s, br, 1 B), –17.5 (s, 1 B), –14.0 (s, 3 B), –13.2 (s, 2 B), –12.3 (s, 2 B), –11.1 (s, 3 B), –9.9 (s, 3 B), –8.5 (s, 1 B), –6.1 (s, br, 2 B), –5.3 (s, 1 B), –2.1 ppm (s, 1 B, BC). ^{11}B NMR ($CDCl_3$): δ = –18.4 (m, 2 B), –15.1 to –8.6 (m, vbr, 14 B), –6.2 (m, br, 3 B), –2.1 ppm (s, 1 B, BC). LRMS (EI): $C_6H_{26}B_{20}O$, m/z calcd: 330.4 ($[M]^+$); found: 330.4; m/z calcd: 312.4 ($[M–H_2O]^+$); found: 312.4.

1-Methyl-7-(1,7-dicarba-*closo*-dodecaboran-9-ylmethyl)-1,7-dicaraba-*closo*-dodecabo-rane (SP2): This compound was isolated as a side product in the synthesis of compound **4** as a colorless crystalline solid (529 mg, 1.68 mmol, 18%, R_f = 0.58, 10:1, ethyl acetate/*n*-hexane, v/v; see synthetic procedure of **4**, main text). Colorless crystals of **SP2** suitable for X-ray structure determination were obtained from $CHCl_3$ at room temperature. Crystallographic data are given in Table S3, and the molecular structure is depicted in Figure S2. Elemental analysis: $C_6H_{26}B_{20}$, calcd (%): C 22.91, H 8.33; found (%): C 23.00, H 8.51. T_m: 99–101°C (*n*-hexane). IR (KBr): ṽ = 3419 (w, H bridges), 3060 (m, $\nu C_{sp2}H$), 2940 (w, $\nu C_{sp3}H$), 2923 (w, $\nu C_{sp3}H$), 2904 (w, $\nu C_{sp3}H$), 2875 (w), 2601 (s, νBH), 1449 (w), 1419 (m), 1387 (w), 1287 (w), 1261 (w), 1191 (w), 1156 (m), 1103 (w), 1068 (m), 1008 (m), 980 (m), 922 (m), 845 (w), 813 (w), 779 (w), 756 (w), 722 (m), 689 (w), 664 (w), 540 (w), 515 (w), 479 (w) cm^{-1}. ^{1}H NMR ($CDCl_3$):

δ = 1.37 – 3.62 (m, vbr, 19 H, $B_{10}H_9$ and $B_{10}H_{10}$), 1.69 (s, 3 H, CH_3), 1.89 (s, br, 2 H, CH_2), 2.89 ppm (s, br, 2 H, 2x$CH_{Cluster}$). $^{13}C\{^1H\}$ NMR ($CDCl_3$): δ = 24.6 (s, CH_3, CH_3), 26.5 (s, br, CH_2, CH_2), 54.0 (s, br, CH, 2x$CH_{Cluster}$), 70.2 (s, C_q, C_qCH_3), 77.3 ppm (s, br, C_q, C_qCH_2). $^{11}B\{^1H\}$ NMR ($CDCl_3$): δ = –19.4 (s, br, 1 B), –17.6 (s, 1 B), –14.0 (s, 2 B), –13.1 (s, 2 B), –12.2 (s, 2 B), –10.3 (m, br, 7 B), –7.1 (s, 2 B), –6.1 (s, br, 2 B), –2.0 ppm (s, 1 B, BC). ^{11}B NMR ($CDCl_3$): δ = –19.4 (d, $^1J_{BH}$ = 178 Hz, 1 B), –17.6 (d, $^1J_{BH}$ = 183 Hz, 1 B), –15.1 to –12.2 (m, br, 6 B), –11.9 to –8.9 (m, br, 7 B), –8.3 to –4.9 (m, br, 4 B), –2.0 ppm (s, 1 B, BC). LRMS (ESI–): $C_6H_{26}B_{20}$, m/z calcd: 313.4 ($[M–H]^–$); found: 313.4.

4. Crystallographic Data of 4, 6, SP1 and SP2

Table S2: Crystallographic data of compounds **4** and **6**.

Parameters	4[a]	6[b]
Empirical formula	$C_8H_{36}B_{30}$	$C_8H_{36}B_{30}S$
Formula weight	456.67	488.73
Temperature	130(2) K	130(2) K
Wavelength (Mo-K_α)	71.073 pm	71.073 pm
Crystal system	Triclinic	Monoclinic
Space group	$P\bar{1}$	$P2_1/n$
Unit cell dimensions	a = 673.76(2) pm	a = 680.62(5) pm
	b = 1114.77(6) pm	b = 1791.54(8) pm
	c = 1780.25(9) pm	c = 2293.8(2) pm
	α = 96.482(4)°	α = 90°
	β = 90.729(4)°	β = 95.315(6)°
	γ = 91.662(3)°	γ = 90°
Volume	1.3278(1) nm^3	2.7850(3) nm^3
Z	2	4
Density (calculated)	1.142 Mg/m^3	1.166 Mg/m^3
Absorption coefficient	0.047 mm^{-1}	0.121 mm^{-1}
F(000)	468	1000
Crystal size	0.40 x 0.20 x 0.02 mm^3	0.40 x 0.30 x 0.02 mm^3
Θ-range for data collection	1.839 to 29.162°	1.783 to 26.875°
Index ranges	–9 ≤ h ≤ 9, –14 ≤ k ≤ 14, –23 ≤ l ≤ 23	–8 ≤ h ≤ 8, –22 ≤ k ≤ 22, –28 ≤ l ≤ 28
Reflections collected	26141	8060
Independent reflections	6381 [R(int) = 0.0558]	8060 [R(int) = 0.0643]
Completeness to theta	100.0 %; θ = 26.38°	100.0 %; θ = 25.35°
Absorption correction	Semi-empirical from equivalents	Semi-empirical from equivalents
Max. and min. transmission	1 and 0.99537	1.00000 and 0.85179
Refinement method	Full-matrix least-squares on F^2	Full-matrix least-squares on F^2
Data / restraints / parameters	6381 / 0 / 487	8060 / 1 / 362
Goodness-of-fit on F^2	1.019	0.970
Final R indices [I>2σ(I)]	R_1 = 0.0615, wR_2 = 0.1311	R_1 = 0.0566, wR_2 = 0.1215
R indices (all data)	R_1 = 0.1007, wR_2 = 0.1507	R_1 = 0.0940, wR_2 = 0.1282
Residual electron density	0.274 and –0.251 e·$Å^{-3}$	0.438 and –0.319 e·$Å^{-3}$
CCDC-number	1945754	1945755

Comments:

[a] All carborane carbon atoms could be localized with a bond length and displacement parameter analysis.

[b] Two-component Twin. Twin law by rows: 1.00 0.00 0.00; 0.00 –1.00 0.00; –0.61 0.00 –1.00. Twin domain ratio 0.341(1):0.659(1). Excluding H(1S1) all hydrogen atoms were calculated on idealized positions. The central carborane unit is slightly disordered with a ratio of 0.938(1):0.062(1). For the minor 0.062(1) disordered part only the S atom was taken into account. All carborane carbon atoms could be localized from the bond length and displacement parameter analysis. Carbon/boron disorder detectable for C4 and B14 with ratio 0.55(2):0.45(2).

Table S3: Crystallographic data of **SP1** and **SP2**.

**Parameters**	**SP1**[a]	**SP2**[b]
**Empirical formula**	$C_6H_{26}B_{20}O$	$C_6H_{26}B_{20}$
**Formula weight**	330.47	314.47
**Temperature**	130(2) K	130(2) K
**Wavelength (Mo-K$_\alpha$)**	71.073 pm	71.073 pm
**Crystal system**	Monoclinic	Monoclinic
**Space group**	$P2_1/c$	$P2_1/n$
**Unit cell dimensions**	a = 684.33(2) pm	a = 1575.43(5) pm
	b = 2726.44(6) pm	b = 673.72(2) pm
	c = 2071.37(4) pm	c = 1788.15(6) pm
	α = 90°	α = 90°
	β = 95.247(2)°	β = 98.191(3)°
	γ = 90°.	γ = 90°
**Volume**	3.8485(2) nm^3	1.8786(1) nm^3
**Z**	8	4
**Density (calculated)**	1.141 Mg/m^3	1.112 Mg/m^3
**Absorption coefficient**	0.052 mm^{-1}	0.046 mm^{-3}
**F(000)**	1360	648
**Crystal size**	0.5 x 0.2 x 0.1 mm^3	0.20 x 0.20 x 0.02 mm^3
**Θ-range for data collection**	1.975 to 32.214°	2.301 to 28.916°
**Index ranges**	–9 ≤ h ≤ 9, –39 ≤ k ≤ 40, –29 ≤ l ≤ 29	–21 ≤ h ≤ 21, –8 ≤ k ≤ 8, –24 ≤ l ≤ 24
**Reflections collected**	58285	15287
**Independent reflections**	12725 [R(int) = 0.0548]	4442 [R(int) = 0.0590]
**Completeness to theta**	100.0 %; θ = 30.51°	99.9 %; θ = 26.38°
**Absorption correction**	Semi-empirical from equivalents	Semi-empirical from equivalents
**Max. and min. transmission**	1.00000 and 0.98063	1.00000 and 0.99445
**Refinement method**	Full-matrix least-squares on F^2	Full-matrix least-squares on F^2
**Data / restraints / parameters**	12725 / 0 / 695	4442 / 6 / 339
**Goodness-of-fit on F^2**	1.035	1.020
**Final R indices [I>2σ(I)]**	R_1 = 0.0533, wR_2 = 0.1195	R_1 = 0.0573, wR_2 = 0.1242
**R indices (all data)**	R_1 = 0.0875, wR_2 = 0.1352	R_1 = 0.1114, wR_2 = 0.1498
**Residual electron density**	0.348 and –0.245 e·Å^{-3}	0.241 and –0.217 e·Å^{-3}
**CCDC-number**	1945756	1945757

Comments:

[a] Carborane carbon atoms localized with bond length and displacement parameter analysis. With OH···O hydrogen donor-acceptor bonds, a tetramer is formed (Figure S1).

[b] All carborane carbon atoms could be located with a bond length and a displacement parameter analysis.

5. References

1. Andrews, J.S.; Zayas, J.; Jones, Jr., Maitland. 9-Iodo-*o*-carborane. *Inorg. Chem.* **1985**, *24*, 3715–3716.
2. Wingen, L.M.; Scholz, M.S. B-Cyanodicarba-*closo*-dodecaboranes: Facile Synthesis and Spectroscopic Features. *Inorg. Chem.* **2016**, *55*, 8274–8276. [doi:10.1021/acs.inorgchem.6b01667]
3. Li, N.; Zeng, F.; Qu, D.; Zhang, J.; Shao, L.; Bai, Y. Synthesis and Characterization of Carborane-containing Polyester with Excellent Thermal and Ultrahigh Char Yield. *J. Appl. Polym. Sci.* **2016**, *133*, 44202 (1–11). [doi:10.1002/app.44202]
4. Zakharkin, L.I. Synthesis and Some Reactions of 1-Halomethyl-*m*-carboranes. *Zh. Obshch. Khim.* **1981**, *51*, 357–361.
